# Injectable and Self-Healing Chitosan Hydrogel Based on Imine Bonds: Design and Therapeutic Applications

**DOI:** 10.3390/ijms19082198

**Published:** 2018-07-27

**Authors:** Yanshuang Xu, Yongsan Li, Qiaomei Chen, Lihua Fu, Lei Tao, Yen Wei

**Affiliations:** 1 The Key Laboratory of Bioorganic Phosphorus Chemistry Biology (Ministry of Education), Department of Chemistry, Tsinghua University, Beijing 10084, China; vincentli945@gmail.com (Y.L.); chenqm1990@126.com (Q.C.); 2 Guangxi Key Laboratory of Petrochemical Resource Processing and Process Intensification Technology, School of Chemistry and Chemical Engineering, Guangxi University, Nanning 530004, China; lhfu@gxu.edu.cn; 3 Department of Chemistry, Center for Nanotechnology and Institute of Biomedical Technology, Chung-Yuan Christian University, Chung-Li 32023, Taiwan

**Keywords:** chitosan, dynamic imine bond, cell therapy, tumor therapy, wound healing

## Abstract

Biological tissues can automatically repair themselves after damage. Examples include skin, muscle, soft tissue, etc. Inspired by these living tissues, numerous self-healing hydrogels have been developed recently. Chitosan-based self-healing hydrogels constructed via dynamic imine bonds have been widely studied due to their simple preparation, good biocompatibility, and automatic reparability under physiological conditions. In this mini-review, we highlighted chitosan-based self-healing hydrogels based on dynamic imine chemistry, and provided an overview of the preparation of these hydrogels and their bioapplications in cell therapy, tumor therapy, and wound healing.

## 1. Introduction

Hydrogels are a family of soft matters which have the three-dimensional polymeric network and contain large proportion of water [1,2,3]. Hydrogels meet the requirements of physiological environment for cell survival (e.g., high water content), thus, numerous applications of hydrogels in the field of bioengineering have been developed, including drug delivery [4,5], cell culture [6,7], and tissue engineering [8,9,10]. Injectable hydrogel is a special class of hydrogel, which can transform from sol to gel in situ upon physical or chemical stimuli, such as the change of temperature [11,12], pH [13,14], or redox property [15,16] of the environment. The injectable hydrogels can be located at a certain position of the body through minimally invasive method. However, the condition for gelation process is usually too harsh to retain the bioactivity of embedded drugs, proteins, and cells [17], and sometimes cannot be realized under physiological conditions. Meanwhile, the gelation time is difficult to control in vivo. Slow gelation leads to diffusion and loss of the delivered species while fast gelation results in non-uniform hydrogel [18].

Self-healing hydrogel is a new type of hydrogel with the automatic healing ability after destruction. Thus, it might be a solution to overcome the as-mentioned drawbacks of the traditional injectable hydrogels. Self-healing hydrogels can be injected as numerous solid fragments, then the fragments coalesce into an integral gel at the target position. This process is well controlled and easily operated, and the environment within the hydrogel keeps stable during the injection and coalescence, which is important for maintaining the activity of the embedded cells and drugs. The self-healing property of this kind of hydrogel is based on dynamic covalent or noncovalent bonds [19]. Recently, increasing efforts are devoted to the design and fabrication of self-healing hydrogels through dynamic covalent bonds because dynamic covalent bonds combine the reversibility of noncovalent bonds and the stability of covalent bonds. As a result, self-healing hydrogels based on phenylboronate ester [20,21], disulfide [22,23], acyhydrazone [24,25], and imine [26,27] have been successfully developed. Among these dynamic covalent bonds, imine bonds can be simply prepared between amino and aldehyde groups under physiological conditions. Hence, self-healing hydrogels based on imine bonds have broad potential biomedical applications.

**Scheme 1:**
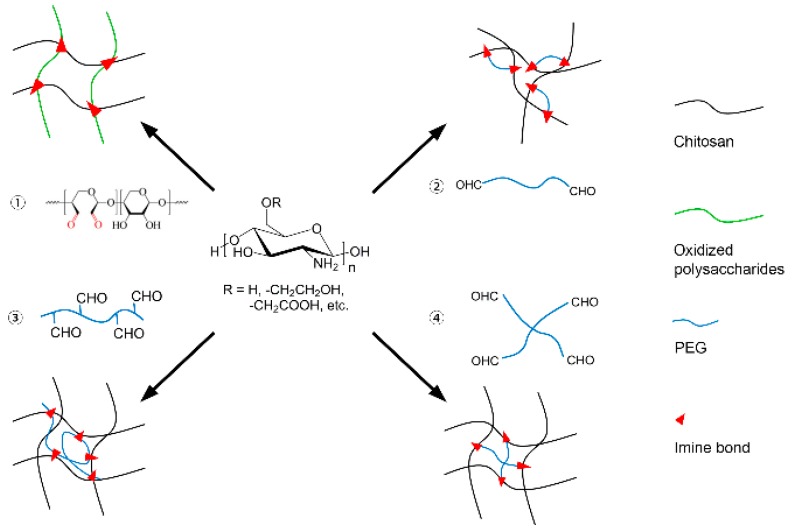
Synthetic strategies of chitosan based hydrogels. Strategy 1: generating aldehyde groups directly on polysaccharides through the oxidation of hydroxymethyl groups; Strategy 2: using linear polymers with two aldehyde groups at the ends of the polymer as the crosslinking agent; Strategy 3: using linear polymers with multiple aldehyde groups as the side chains of the polymer as the crosslinking agent; Strategy 4: using star polymers with aldehyde group at the end of each arm of the polymer as the crosslinking agent.

Chitosan is a natural cationic polymer composed of glucosamine and *N*-acetyl glucosamine. Chitosan has been broadly used in the pharmaceutical and medical fields because of its biocompatibility and biodegradability [28,29]. Therefore, chitosan is an excellent choice to form injectable self-healing hydrogels based on imine bonds owing to the abundant amino groups on its backbone. The key question is to find the proper cross-linking agents with aldehyde groups. Scheme 1 summarized the synthetic strategies for chitosan-based hydrogels. One strategy is to directly generate aldehyde groups on polysaccharides as the cross-linking points through oxidization of hydroxymethyl groups (strategy 1 in Scheme 1). However, strong oxidants, such as NaIO_4_, are necessary for this conversion, which may cause undesired degradation of the polysaccharide backbone [30]. Small dialdehyde molecules can act as the cross-linkers, but their toxicity limits the biological applications of the hydrogels [31]. Comparatively, polymeric di- or multi- aldehydes, especially those based on polyethylene glycol (PEG), show low toxicity. As shown in Scheme 1, polymers with aldehyde groups at the chain ends or as the side chains have been developed as the crosslinking agents. By using different types of crosslinking agents, the mechanical, self-healing and degradation properties of the hydrogels can be tuned. This mini review summarized the recent progresses of chitosan-based injectable and self-healing hydrogels with polymeric di- and multi- aldehydes as cross-linkers, including their preparation and applications in cell therapy, tumor therapy, and wound healing.

## 2. Design and Preparation

PEG has been broadly used in biomedical areas because of its reliable safety, excellent stability, biocompatibility, and hydrophilicity. For example, PEG has been linked with drugs and proteins to notably reduce the immunogenicity and improve the circulation half-life of pharmaceutics in the body [32,33]. Nowadays, many PEG derivatives have been employed as starting materials to synthesize polymeric materials. Therefore, PEG derivatives decorated by aldehyde groups are excellent candidates as the cross-linkers in chitosan-based self-healing hydrogels.

**Figure 1:**
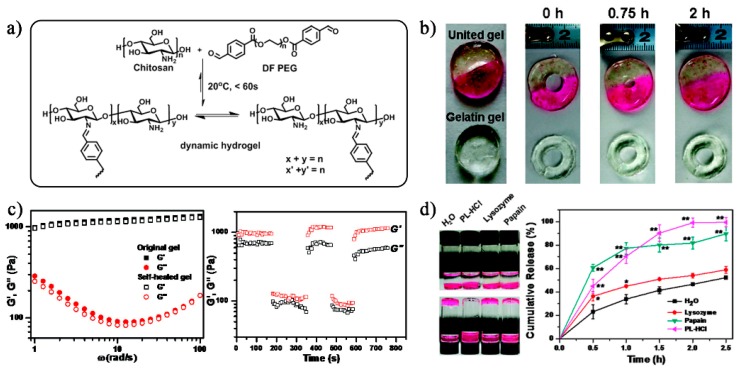
(**a**) The schematic illustration of the dynamic hydrogel; (**b**) the self-healing process between two different colored semicircle hydrogels; (**c**) **left**: storage modulus G’ (black squares) and loss modulus G’’ (red ciecles) of the original (solid) and the self-healed (hollow) hydrogel; **right**: G’ (red hollow squares) and G’’ (black hollow suqares) in continuous step strain experiments; and (**d**) the photos and the plots of cumulative release of rhodamine B under different conditions. Reprinted with permission [34]. Copyright 2011, American Chemical Society.

In 2011, Zhang et al. reported a difunctionalized PEG (DF PEG) as a crosslinking agent to form the hydrogel with chitosan (strategy 2 in Scheme 1), as shown in Figure 1a [34]. DF PEG was achieved via esterification of hydroxyl-terminated PEG with 4-formylbenzoic acid. By mixing the solutions of chitosan and DF PEG, a hydrogel was formed within 60 s at room temperature. The amine groups on the chitosan reacted with the benzaldehyde groups at the end of PEG chains to form imine linkages, leading to the formation of a crosslinking network. Since the aromatic imine bond has better stability than the aliphatic counterparts, hydrogel with DF PEG as the cross-linking agent exhibited improved mechanical property. Meanwhile, the reversible disassociation and regeneration of imine bonds networks endowed the hydrogel with self-healing ability.

The self-healing properties of the hydrogel were further demonstrated qualitatively and quantitatively. As shown in Figure 1b, two semicircle hydrogels (colorless and red, respectively) were contacted intimately to form one piece of the hydrogel. A hole with the diameter of 0.9 cm was punched at the center of the hydrogel. The hole diminished gradually and healed completely after 2 h. As a comparison, a gelatin gel did not show the self-healing ability. Moreover, rheology experiments showed that the storage modulus G’ and loss modulus G’’ values of this hydrogel after self-healing test reached the same level as that of the virgin hydrogel, as shown in Figure 1c, indicating the reconstructing of the inner network. Then continuous step-change oscillatory strain between 200% and 20% at the same frequency of 1 Hz was also applied to the hydrogel (Figure 1c (right)). The G’ value showed an alternative decrease and increase under high strain and low strain, respectively. This indicated that the network of this chitosan-based self-healing hydrogel was damaged by high strain and quickly restored under low strain. In addition, chemical or biological stimuli affected the imine bonds, leading to the deconstruction of the hydrogel (Figure 1d). These stimuli included a change of pH, amino acids (with amino groups), and vitamin B6 derivatives (with aldehyde groups). Therefore, rhodamine B and lysozyme were used as models of small drugs and biomacromolecules, and released from the hydrogel under control respectively based on the multi-responsibility of the hydrogel.

**Figure 2:**
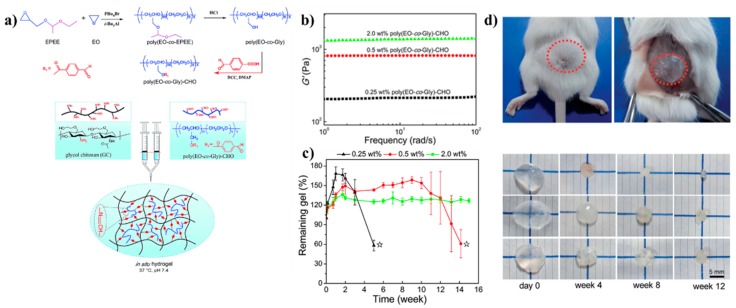
(**a**) Schematic representation of the formation of the hydrogel cross-linked by a Schiff base reaction between GC and poly(EO-*co*-Gly)-CHO; (**b**) G’ of the hydrogels containing different concentrations of poly(EO-*co*-Gly)-CHO depending on the frequency. The final concentration of GC was fixed at 1.5 wt %; (**c**) in vitro degradation of GC/poly(EO-*co*-Gly)-CHO hydrogels fabricated with different concentrations of cross-linker in PBS containing 1 mg mL^−1^ lysozyme at 37 °C; (**d**) in vivo degradation tests of the hydrogels. Photographs of the remaining hydrogels containing the indicated concentrations of poly(EO-*co*-Gly)-CHO: 0.25 wt % (**top**), 0.5 wt % (**medium**), and 2.0 wt % (**bottom**) removed from ICR mice after 0–12 weeks. Reprinted with permission [35]. Copyright 2015, Royal Society of Chemistry.

Furthermore, multi-aldehyde molecules have more possible cross-linking points to chitosan to improve the effectiveness and tunability of the gelation of the hydrogel (strategy 3 in Scheme 1). For instance, Cao et al. used multi-aldehyde functionalized PEG as a cross-linker to react with chitosan derivative to form the injectable hydrogel, as presented in Figure 2a [35]. First, the random copolymer poly(EO-*co*-EPEE) was synthesized by anionic polymerization. After deprotection, the benzaldehyde unit was introduced to poly(EO-*co*-Gly) through the reaction between the free hydroxymethyl groups and 4-formybenzoic acid. The obtained poly(EO-*co*-Gly)-CHO still maintained good water solubility, which was indispensable for the subsequent biological applications. In a typical injection process, the solutions of glycol chitosan (GC) and poly(EO-*co*-Gly)-CHO with the equivalent volumes were mixed at the “Y” shape connector and the dynamic hydrogel formed spontaneously. The mechanical strength of the GC/poly(EO-*co*-Gly)-CHO hydrogels can be tuned in a wide range with control simply by changing the concentration of the cross-linker, as illustrated in Figure 2b. The tunability of the mechanical property endows this hydrogel with more possibility in regulating cellular function in substrates with different stiffness.

In addition to the mechanical strength, the biodegradation rate of this hydrogel can also be tuned by changing the concentration of the multi-aldehyde cross-linker. Both of the in vitro and in vivo degradation experiments showed that the GC/poly(EO-*co*-Gly)-CHO hydrogels degraded faster as the concentration of poly(EO-*co*-Gly)-CHO decreased. In the in vitro experiment, as shown in Figure 2c, in PBS containing 1 mg·mL^−1^ of lysozyme, the hydrogels fabricated with 0.25 wt % and 0.5 wt % of poly(EO-*co*-Gly)-CHO collapsed at 5 and 14 weeks, respectively. While the one containing 2.0 wt % of cross-linker did not show any degradation even after 15 weeks, due to the intact inner structure in the hydrogel. Furthermore, the GC/poly(EO-*co*-Gly)-CHO hydrogels were implanted into the backs of ICR mice to investigate degradation in vivo, as shown in Figure 2d. Similarly, after the same period of time, the size of the residual hydrogel was smaller as the concentration of the cross-linker in the hydrogel was lower. The degradability of biomaterials is a prerequisite of their safe and non-invasive elimination from the body, and the degradation creates space for cell growth and for the newly-formed tissues. The degradation rates of the GC/poly(EO-*co*-Gly)-CHO can be well controlled, and the generated byproducts from degradation had outstanding biocompatibility, low immunological response, and low cytotoxicity. The degradation property of this hydrogel is a key advantage for biological applications in living body.

**Figure 3:**
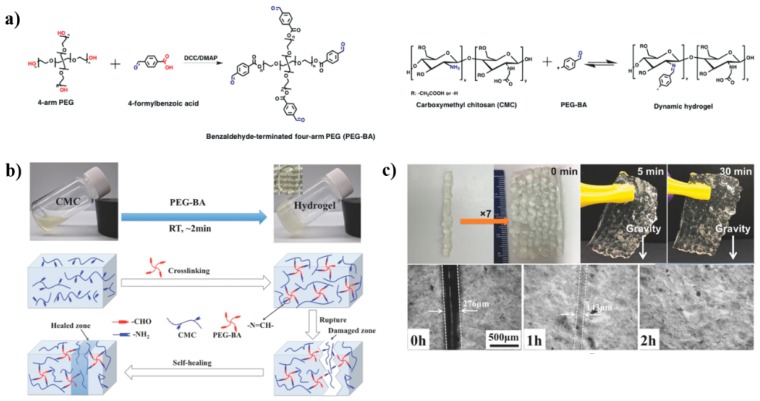
(**a**) the synthesis scheme of benzaldehyde-terminated four-arm PEG (PEG-BA), and PEG-BA conjugated amino group of CMC to form dynamic hydrogel; (**b**) schematic illustration of the gelation and self-healing process; (**c**) macroscopic self-healing process of the hydrogel. **Top**: the hydrogel strips extruded from syringe and healed to a whole piece and suspended under gravity; **bottom**: optical microscope images of hydrogels after healing for 0, 1, and 2 h at room temperature. Reprinted with permission [36]. Copyright 2016, Wiley-VCH.

In addition, star-like PEG is an appropriate choice to play the role of the cross-linking agent (strategy 4 in Scheme 1). Compared with linear PEG, branched PEG is an impenetrable space-filled sphere and there are much fewer entanglements frozen in the network. Therefore, the branched PEG can be employed to form strong hydrogels. Based on above advantages, benzaldehyde-terminated telechelic four-armed PEG (PEG-BA) and carboxymethyl chitosan (CMC) were used to prepare the dynamic hydrogels with high mechanical performance and fast self-healing ability, as reported by Huang et al. [36]. In details, water-soluble CMC was synthesized through the reaction between chitosan and monochloroacetic acid under alkaline condition. PEG-BA was produced by conjugating 4-formybenzoic acid to the terminal hydroxyl of four-arm PEG via carbodiimide coupling reaction similar to the preparation of DF PEG, as presented in Figure 3a.

The nature of imine bonds rendered the CMC/PEG-BA hydrogels with outstanding self-healing performance. Figure 3c showed the macroscopic healing process. First, the strip-to-film experiment was used to assess the self-healing ability of the hydrogels. The hydrogel strips were extruded from the syringe and placed onto a PTFE plate side by side. After 5 min, the strips integrated into a whole film without any external intervention. No cracks formed between strips due to gravity when the hydrogel was suspended, indicating high self-healing efficiency and mechanical strength. Additionally, the self-healing process of the cut surfaces was monitored by optical microscopy. Interestingly, a crack of the width of 276 μm between the two cut surfaces completely disappeared after 2 h. During the healing process, a “mobile phase” was necessary at the damaged interface, where the imine bonds cleaved and the free aldehyde and amine groups exposed. The newly-formed imine bonds connected the cut surfaces together. This crack healing experiment revealed that dynamic reaction rather than the simple adhesion took place at the interfaces of broken hydrogels.

## 3. Biological Applications

Over the past few decades, hydrogels have revolutionized the biomedical field and have been employed in a wide variety of applications, such as controlled drug delivery system, cell culture supports, and tissue engineering supports. Due to their high water content and moderate mechanical properties, hydrogels are the most promising candidates to mimic the native extracellular matrix (ECM) for tissue regeneration. The self-healing hydrogels based on chitosan and PEG derivatives are highly biocompatible, which is important to maintain the viability and functions of the encapsulated cells, drugs, and proteins. As summarized by the previous section, the mechanical strength of this type of hydrogels is relatively low (almost in the magnitude of kPa) due to the dynamic nature of the imine bonds, limiting their applications in some fields, such as bone repairing. However, the moderate mechanical strength is suitable for injection. Therefore, these type of hydrogels are ideal carriers to deliver the biological substance to the injection spots without un-controlled leakage. Various type of species including molecules, cells, and nanomaterials can be delivered simultaneously. Hence, self-healing hydrogels provide the opportunities for the combination of different treatments to improve the therapeutic effect. Furthermore, the self-healing hydrogels can also be used as wound dressing due to the fit-to-shape property for any defect. In this section, we will focus on the recent progress in the applications of the self-healing hydrogels, such as cell therapy (repairing of the central nervous system, blood capillary, etc.), tumor therapy, and wound healing.

### 3.1. Cell Therapy

Since drug therapy failed in some diseases like leukemia and cartilage disorder, many efforts have been shifted towards cell therapy, which might be a more direct approach to achieve the ideal therapeutic purpose. However, how to transplant the cells to the injured sites and maintain their biological activities and functions is still a difficult problem. One possible solution is to incorporate cells into protective biomaterials. Hydrogels, due to their good biocompatibility and similarity to ECM, can serve as promising candidates for cell delivery. Using self-healing hydrogels as the cell carrier can protect the embedded cells from being rejected by the body and anchor high concentrated cells at the target sites to improve the therapeutic efficacy [37]. On the other hand, self-healing hydrogels can be directly injected into the damaged position without surgical operation. Moreover, thanks to the reversible linkages of the hydrogels, the embedded cells can adjust their morphology, spread, and migrate [38].

**Figure 4:**
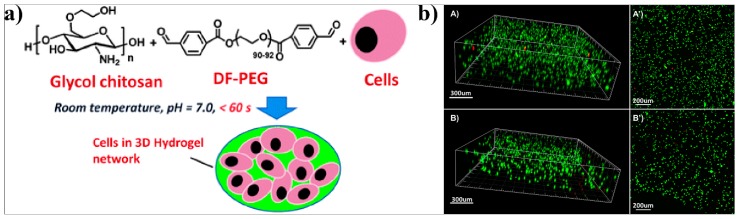
(**a**) Preparation of cells encapsulated in GC/DF PEG hydrogels under physical conditions; and (**b**) cell viability (viable cells: green; dead cells: red) and spatial distribution of Hela cells embedded in the hydrogel after 24 h and 72 h. Reprinted with permission [39]. Copyright 2012, Royal Society of Chemistry.

To function effectively as a 3D cell-culture scaffold, the self-healing hydrogels need to satisfy the conventional basic requirements for ECM mimetic materials. Therefore, the gel precursors need to be biocompatible, and the rate of the gel crosslinking reaction need to be proper for cell encapsulation without sedimentation. In 2012, Yang et al. reported 3D encapsulation of cells in the GC/DF PEG hydrogel for cell culture [39]. First, the hydrogel precursors showed good biocompatibility. The cytotoxicity test of the synthesized DF PEG was carried out by using Hela cells. The viability examination showed no obvious viability decrease, indicating excellent biocompatibility of DF PEG. GC, the other component of this hydrogel, is a well-known biocompatible polymer. Therefore, this hydrogel was almost non-toxic. More importantly, the implanted process was very simple and mild. As presented in Figure 4a, by mixing the GC solution, cell suspension and DF PEG solution, the hydrogel containing cells was obtained. As revealed by dying and confocal microscopy images, the viability of HeLa cells in the 3D encapsulation was 97% and 87% after 24 and 72 h culture, respectively. This result indicated that the hydrogel played the roles not only as ECM matrix, but also as a porter of nutrients and oxygen to cells. Furthermore, due to the shear-thinning and self-healing properties of GC/DF PEG hydrogels, the hydrogels with implanted HeLa cells were injected into a Petri dish and the cells retained high viability after injection. Therefore, this is a promising system for the delivery of cells. Besides, the hydrogel with PEG based multi-aldehyde as cross-linker also showed good biocompatibility. For instance, chondrocytes in GC/poly(EO-*co*-Gly)-CHO exhibited a significant proliferation after 14 days of 3D culture [35].

**Figure 5:**
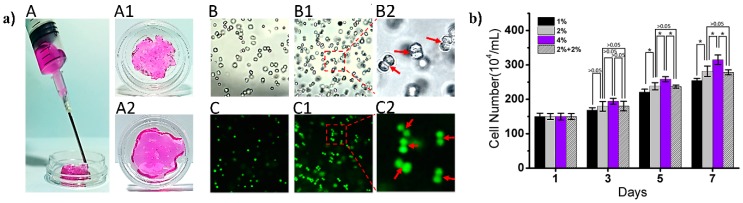
(**a**) Injection and self-healing of the cell-laden GC/DF PEG hydrogel (A, A1 and A2), and the optical microscopy (B and B1) and fluorescent images (C and C1) of the embedded cells on 1st and 7th day culture, respectively. B2 and C2 are the 4 times magnification of the red squares in image B1 and C1, respectively. The red arrows indicate the cell divisions; (**b**) cell numbers in a series of hydrogels with different mass fractions of DF-PEG at denoted time after injection. ‘2% + 2%’ refers to a hydrogel containing 2% DF-PEG and 2% PEG without aldehyde groups, which contained the same mass of PEG as the hard hydrogel (4%), while the modulus was similar to that of the medium hydrogel (2%). Data represent mean ± SD (*n* = 3, *: *p* < 0.05). Reprinted with permission [40]. Copyright 2017, Elsevier Ltd.

Additionally, the mechanical performance of the hydrogel has been found to be important in cell proliferation. Li et al. studied the influence of stiffness of the GC/DF PEG hydrogel on the cell proliferation [40]. By tuning the concentration of DF PEG, a series of hydrogels with different mechanical strength were prepared, which were classified into three types, namely soft (1% DF PEG), medium (2% DF PEG) and stiff (4% DF PEG) hydrogels. Interestingly, the strength of the stiff hydrogel was similar to that of the in vivo environment of L929 cells, which was advantageous to acquire the highest cell proliferation rate. To mimic the procedures of cell therapy, the injection and the followed post-culture experiments were performed. In detail, the cell-laden hydrogel was squeezed out through a syringe, and the squished pieces reformed an integrated hydrogel due to its excellent self-healing ability, as shown in Figure 5a. The density of cells increased significantly during the culture for seven days. More impressively, cell division was observed in the magnified images. Furthermore, the quantitative statistical analysis indicated that the cell proliferation showed the strong dependence on the stiffness of hydrogels (Figure 5b): the cell number increased 70%, 88%, and 110% in the soft, medium, and stiff hydrogels, respectively. For the deep understanding of the correlation between the stiffness of hydrogels and the proliferation of cells, more issues need to explore, such as the influence of the stiffness on the cell metabolism and the interaction among cells.

**Figure 6:**
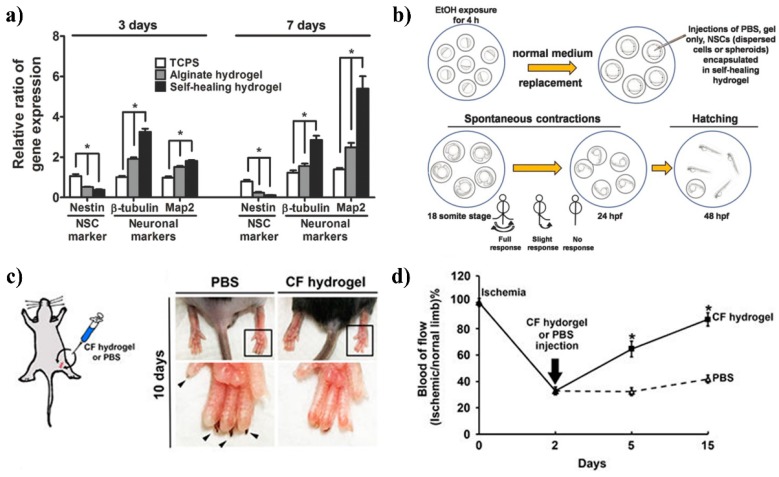
(**a**) The expressions of specific neuronal-related genes in the TCPS, alginate hydrogel and self-healing hydrogel; (**b**) the treatment procedure for central nervous system rescue in zebrafish by the self-healing hydrogel. Firstly, ethanol exposure of the developmental zebrafish embryos resulted in the central nervous system deficits. The medium was then replaced with fresh normal medium following various treatments including PBS, NSC spheroid-laden hydrogel, NSC-laden hydrogel, only hydrogel, and NSC dispersed hydrogel. The spontaneous contraction of the embryos examined at 18 somite stage. Reprinted with permission [42]. Copyright 2015, Wiley-VCH; (**c**) the left femoral artery and iliac artery in mice were cut to induce hindlimb ischemia. The right part shows representative images of mouse ischemic hindlimbs after injection with PBS or CF hydrogel. The arrow indicated nail loss; and (**d**) the significant blood flow increased on 5 and 15 days post-surgery in the CF hydrogel-injected group much higher than those in the PBS-injected group. Reprinted with permission [43]. Copyright 2017, Nature Publishing Group.

Beyond cell proliferation, other cell functions can also be adjusted by tuning the stiffness of the hydrogels. For example, the differentiation of neural stem cells (NSCs) is modulated by the substrate stiffness [41]. In relatively soft materials (0.1–1 kPa), most of NSCs differentiate to neurons, while in a slightly stiffer material (7–10 kPa), differentiate to glial cells. Therefore, it is possible to control the directions of differentiation by simply tuning the mechanical properties of the substrates. As reported by Tseng et al., NSCs were introduced to heal the damaged central nervous system of zebrafish by using the GC/DF PEG hydrogel as a substrate [42]. In this experiment, murine NSCs encapsulated into self-healing hydrogels expressed more neuronal marker genes compared with ones in the traditional culture plate and in alginate hydrogels, as presented in Figure 6a. The neurosphere-like NSC aggregates showed faster proliferation and higher differentiation tendency than neuron-like cells, probably because the aggregation increased cell-cell contact to own better multipotency and secreted more cytokines to enhance the migration and survival of cells. The zebrafish was used as a model to evaluate the therapeutic effects. Figure 6b illustrated the treatment procedures. Several groups of damaged embryos were injected with NSC spheroid-laden hydrogel, NSC-laden hydrogel, only hydrogel, and NSC dispersed hydrogel, respectively. Among them, the group injected with NSC spheroid-laden hydrogel exhibited the highest coiling frequency and hatching rate, approximating the wild-type control, followed by the group injected with NSC-laden hydrogel. It was noteworthy that the group injected with solely the hydrogel showed a better recovery efficiency than the group injected with dispersed NSCs. A probable reason was that the primary amine groups on the chitosan backbone interacted with NSC progenitors and further induced the neuronal differentiation, while the dispersed NSC had low cell survival. Therefore, GC/DF PEG hydrogel not only acts as an extracellular matrix for the proliferation and differentiation of the embedded cells, but also promotes the function of cells.

Furthermore, bioactive molecules are introduced into hydrogels to develop new functional materials. Fibrin, a biological polymer in vertebrates, can adhere to the arterial endothelial cell (EC) and induce angiogenesis at the injured sites. A composite self-healing hydrogel based on chitosan, fibrin, thrombin, and DF PEG was established by Heish et al. and named as CF hydrogel [43]. The formation of an interpenetrating polymer network (IPN) between chitosan and fibrin endowed the CF hydrogel with fast self-healing, slow degradation and capillary formation capabilities. The injection of CF hydrogel into the perivitelline space of zebrafish embryos induced an angiogenic response in vivo. Moreover, the test of recovery of mouse hindlimb ischemia confirmed the ability of hydrogels to induce blood capillary formation (Figure 6c). Figure 6d compared the recovery of blood flow at 3 and 13 days after injection with CF hydrogel. However, angiogenesis is a complex process, accompanied by the risk of causing cancer unexpectedly. This work demonstrated the possibility of the application of CF in angiogenesis, and more efforts are needed to reduce the risk of the treatment and improve the clinical performance of this hydrogel.

### 3.2. Tumor Therapy

Chemotherapy is a common method for clinical anti-tumor in which chemical drugs are used to kill tumor cells and inhibit the growth and proliferation of tumor cells. Although great success has been achieved, chemotherapy still faces some limitations. Chemotherapy drugs, either oral or injected intravenously, reach disease sites through the long path in the circulatory system. High oral dose or high frequency of injection is usually necessary due to the dilution of the drugs in blood or body fluid, which causes inevitable side effects on the normal cells and makes the patients suffer seriously. To solve these problems, one strategy is to apply the drugs directly to the disease sites to avoid long circulation. Carriers of drugs are indispensable in order to prevent the leakage of the drugs from tumor surroundings. The appropriate carriers need to meet some criteria, including biocompatibility, degradability, injectability, and stability. Additionally, the carriers must possess the proper mechanical strength to hold the drugs at one place and the ability to release the drugs controllably. Based on the as-mentioned requirements, self-healing hydrogels are the potential candidates as the drug delivery vehicles. Strategies for using self-healing hydrogels for tumor ablation have been designed and developed to achieve admirable therapeutic effects.

**Figure 7:**
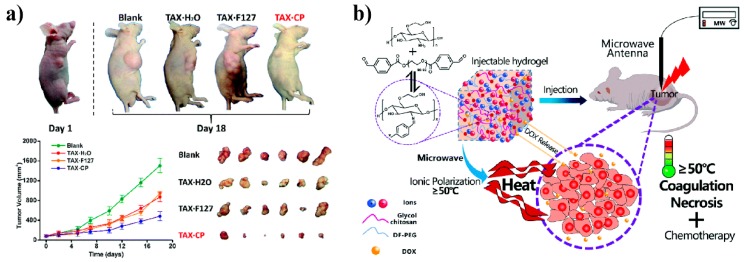
(**a**) The results of the intra-tumor injection experiments. The nude mice were divided into four groups based on different treatments, namely blank group (injection only with saline water), TAX·H_2_O group (injection with Taxol solution), TAX·F127 group (injection with Taxol in pluronic F127 hydrogel) and TAX·CP group (injection with Taxol in CP hydrogel). The bottom part shows the relative tumor volume changes under various treatments. Reprinted with permission [44]. Copyright 2017, Royal Society of Chemistry; and (**b**) a schematic illustration of microwave ablation therapy. The ionic hydrogel encapsulating DOX was injected in the tumor site of the mice. Under microwave irradiation, the hydrogel induced a rapid and remarkable temperature increase from room temperature to 50 °C, accompanied by a constant release of DOX. Reprinted with permission [45]. Copyright 2017, Royal Society of Chemistry.

As reported by Yang et al., the GC/DF PEG hydrogel containing Taxol (referred as TAX-CP) was used for tumor chemotherapy [44]. The release of Taxol from TAX-CP was significantly slower than from F127 hydrogel and the cumulative release increased steadily over seven days, indicating that the self-healing hydrogel provides good encapsulation and constant release of drugs. Subsequently, the in vivo injection experiment was conducted on the tumor-bearing nude mice. Figure 7a displayed the growth of tumors after different treatments. Compared with the treatment of TAX with water and TAX with F127, the treatment of TAX-CP led to the more significant decrease in the volume of the tumor. The tumor growth inhibition (TGI) of TAX-CP treatment was more than two times of those of TAX-H_2_O and TAX-F127 treatments, suggesting the outstanding therapy effect. This was attributed to that GC/DF PEG hydrogel, with good self-healing ability and stability, confined the diffusion and reduced the loss of TAX.

Different kinds of substances can be loaded into the self-healing hydrogels and injected simultaneously. Recently, self-healing hydrogels with multiple encapsulated components are being developed to combine two or more treatment methods to improve the therapeutic effects of tumors. For instance, Wang et al. developed the di-functional GC/DF PEG hydrogel containing doxorubicin hydrochloride (DOX) and high concentration of salt for combining chemotherapy and microwave tumor ablation [45]. Microwave tumor ablation is a technique to generate heat fast localized in the tumor, with the advantage of large-volume tumor ablation, deep penetration in tissue and less pain to patients [46]. The high porosity of the hydrogel provides the confinement to a large number of ions. Hydrogels containing a high concentration of salt, which show strong polarization and rapid heat generation under microwave irradiation, can serve as a carrier for microwave tumor ablation. The chicken breast was used as a model tissue to investigate the heat effect. Under microwave with ultralow power, the temperature increasing was localized in the ionic hydrogel without causing overheating of the surrounding tissues, suggesting this heat effect can kill tumor cells without damaging healthy tissues. Figure 7b showed the treatment process for tumor therapy in mice. Under microwave interaction, the local temperature in the tumor area increased rapidly to 50 °C. This localized high temperature can, on one hand, ablate tumor cells and, on the other hand, promote the release of DOX, leading to good therapy efficiency. Therefore, this kind of bifunctional ionic hydrogels with the abilities of microwave ablation and drug delivery open the new door to tumor therapy.

**Figure 8:**
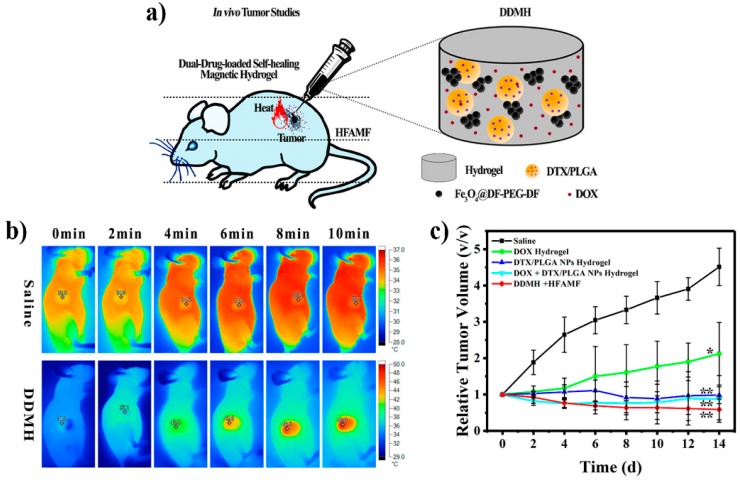
(**a**) Schematic illustration of dual-drug-loaded magnetic hydrogel (DDMH) for cancer treatment; (**b**) infrared thermal image for tumor-bearing mice exposed to an AMF (19.99 KA/m) for 10 min at post-injection of saline and DDMH (100 μL); and (**c**) tumor growth curves with different treatments (saline group, DOX group, DTX/PLGA NPs group, DOX + DTX/PLGA NPs group, and DDMH + AMF group). Data presented the mean ± standard deviation of five mice. Reprinted with permission [47]. Copyright 2015, American Chemical Society.

To achieve the synergetic therapeutic effect of multiple components, the engineering of multi-functional composite self-healing hydrogels is delicate. Recently, Xie et al. designed a thermosensitive dual-drug-loaded magnetic GC/DF-PEG hydrogel (DDMH), as shown in Figure 8a [47]. This hydrogel encapsulated both DOX and docetaxel (DTX)-PLGA nanoparticles for chemotherapy. Iron oxide nanoparticles were also embedded to efficiently convert alternative magnetic field (AMF) into localized heat as the stimuli for drug release. Since DTX was encapsulated in PLGA nanoparticles, it released later than DOX. However, once the releasing process of DTX started, both the release rate of DOX and DTX was found to increase dramatically thanks to the synergistic effect between them. Moreover, the release rate of DTX from the PLGA nanoparticles was accelerated under AMF treatment, ascribed to the higher temperature induced a fast diffusion rate of DTX, as well as the biodegradation rate of PLGA. These special release behaviors are promising to overcome the multidrug resistance for tumor chemotherapy. The antitumor effect was further evaluated by using MDA-MB-231 tumor-bearing nude mice as the model animal. As shown in Figure 8b, the temperature of the tumor injected with DDMH increased dramatically upon AMF exposure, which triggered the release of DTX in the co-delivery system. Furthermore, in the in vivo antitumor assays in Figure 8c, the group injected with DDMH which released DOX and DTX controllably triggered by AMF showed more remarkably decrease in tumor volume compared with other control groups. Thus, the DDMH is a potential new carrier with asynchronous control release of multiagent in the cancer treatment.

### 3.3. Wound Healing

**Figure 9:**
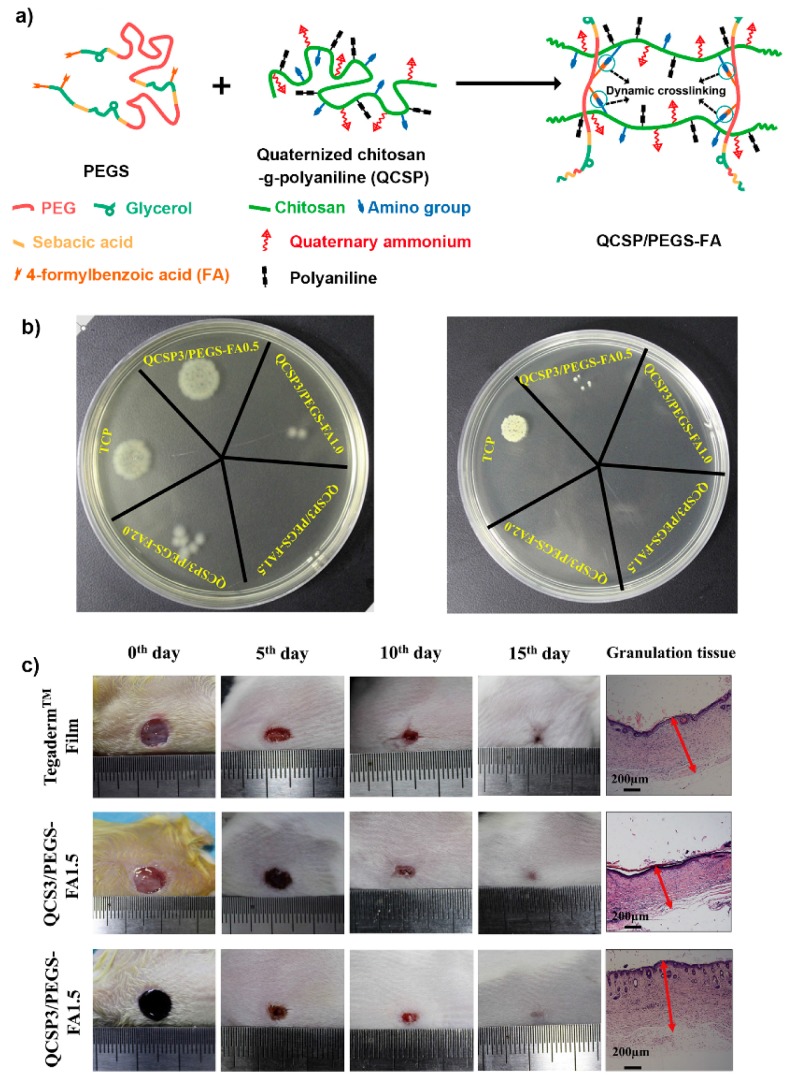
(**a**) The schematic illustration of the dynamic hydrogel; (**b**) photographs of survival bacteria, *E. coli* (**left**) and *S. aureus* (**right**), clones on agar plates after contacting with hydrogels: (**c**) photographs of wounds at the 0th, 5th, 10th, and 15th day and granulation tissue at the 15th day for commercial film dressing (Tegaderm™), hydrogel QCS3/PEGS-FA1.5 and hydrogel QCSP3/PEGS-FA1.5. Among all groups, the hydrogel QCSP3/PEGS-FA1.5 treated wound showed the smallest area and thickest granulation tissue. Reprinted with permission [50]. Copyright 2017, Elsevier Ltd.

Skin, as the largest organ in the body, is vulnerable to external attacks. Various commercially available dressings, such as polyurethane films, fibers, and hydrogels, are already used for the treatment of wounds [48]. Hydrogels as the wound dressing have a lot of inherent advantages, such as providing moist environments, absorbing wound exudates, allowing oxygen to permeate and cooling the wound surfaces [49]. Especially, the injectable and self-healing hydrogels have received plenty of attention because they can fill the irregular shapes of wounds and tolerate the deformation or damage caused by external force. Additionally, wound dressings with good antibacterial properties can prevent wounds from infection. Chitosan has inherent antibacterial activity because the amino group can turn into ammonium group in the acid environment. However, this antibiotic property is limited in the non-acidic environment [50]. While the quaternized chitosan is an excellent antibacterial material under any condition. Zhao et al. developed a series of quaternized chitosan-based injectable and self-healing hydrogels (Figure 9a, referred as QCSP/PEGS-FA hydrogel, the number after the copolymer name meant the mass concentration of component) for wound healing [51]. In this series of hydrogels, polyaniline was grafted onto the quaternized chitosan backbone to further improve the electroactivity and enhance antibacterial capacity. In the surface antibacterial experiment, the hydrogels with the mass fraction of cross-linker higher than 0.5 showed the good performance to kill the *E. coli* and *S. aureus*, as shown in Figure 9b. Then, the wound healing test in vivo using a full-thickness skin defect model was investigated. The QCSP3/PEGS-FA1.5 hydrogel showed the significantly better effect of wound healing than the commercial film dressing due to the synergistic effect between quaternized chitosan and polyaniline. In addition, the chitosan-based hydrogels encapsulated antibacterial drugs or polyamino acid are excellent candidates as wound dressings.

## 4. Summary and Outlook

In recent decades, the development of self-healing hydrogels constructed by dynamic covalent bonds has been booming. Schiff base, as one of dynamic covalent bonds, is explored extensively to prepare hydrogels for medical applications because its decoupling and recoupling can occur under mild conditions without any stimuli. In this mini review, we specially focused on self-healing hydrogels obtained from the derivatives of chitosan and PEG, and introduce their synthetic strategies, mechanical properties and biological applications in cell therapy, tumor therapy, and wound healing. Thanks to the fast reaction kinetics of imine bonds, the hydrogel can integrate as a whole after injection, which provides the possibility for minimally invasive methods. Additionally, the deformability capacity also plays an important role in adapting the irregular shapes to benefit wound healing.

However, the self-healing chitosan hydrogels based on imine bond still have some limitations. For example, since chitosan and its derivatives are derived from shrimp and crabs, a large number of procedures are required to remove the trace amounts of proteins which may cause an immune response in clinical application. Furthermore, due to the nature of dynamic covalent bonds and relatively low solubility of chitosan, the self-healing hydrogels usually show the relatively low mechanical properties, limiting their applications in some therapies such as bone repairing. Moreover, the degradation rate is relatively fast and it is difficult to meet the requirements of some tissue engineering.

To overcome the above problems, one strategy is to develop a new method to obtain chitosan and its derivatives with good biocompatibility or to seek other available compounds to replace chitosan. Additionally, deep understanding of the mechanism of self-healing is significant for the preparation and the optimization of self-healing hydrogels. It is needed to fill the gap between theory and experiment through computer simulation. Moreover, it is of great significance to develop multi-component self-healing hydrogels to fulfill various functions. The smart hydrogels, which integrate self-healing ability and other capabilities, such as electrical conductivity, magnetism, and luminescence into a single hydrogel system, have potential applications in an artificial electronic skin, soft actuator, and artificial muscle.

## Abbreviations

PEG	Polyethylene glycol
GC	Glycol chitosan
CMC	Carboxymethyl chitosan
ECM	Extracellular matrix
NSC	Neural stem cell
EC	Endothelial cell
IPN	Interpenetrating polymer network
TGI	Tumor growth inhibition
DOX	Doxorubicin hydrochloride
DTX	Docetaxel
DDMH	Dual-drug-loaded magnetic
